# Monocrotaline-Induced Pulmonary Arterial Hypertension and Bosentan Treatment in Rats: Focus on Plasma and Erythrocyte Parameters

**DOI:** 10.3390/ph15101227

**Published:** 2022-10-05

**Authors:** Tomas Jasenovec, Dominika Radosinska, Marta Kollarova, Norbert Vrbjar, Peter Balis, Simona Trubacova, Ludovit Paulis, Lubomira Tothova, Ivana Shawkatova, Jana Radosinska

**Affiliations:** 1 Institute of Physiology, Faculty of Medicine, Comenius University in Bratislava, 813 72 Bratislava, Slovakia; 2 Institute of Immunology, Faculty of Medicine, Comenius University in Bratislava, 811 08 Bratislava, Slovakia; 3 Premedix Academy, 811 02 Bratislava, Slovakia; 4 Centre of Experimental Medicine, Slovak Academy of Sciences, 841 04 Bratislava, Slovakia; 5 Institute of Pathophysiology, Faculty of Medicine, Comenius University in Bratislava, 811 08 Bratislava, Slovakia; 6 Institute of Molecular Biomedicine, Faculty of Medicine, Comenius University in Bratislava, 811 08 Bratislava, Slovakia

**Keywords:** pulmonary arterial hypertension, monocrotaline, bosentan, angiotensins, oxidative stress, erythrocytes

## Abstract

The objective of our study was to contribute to the characterization of monocrotaline-induced pulmonary arterial hypertension (PAH) in a rat model, with emphasis on the renin–angiotensin–aldosterone system, parameters of oxidative stress, the activity of matrix metalloproteinases, and erythrocyte parameters. Moreover, we aimed to analyze the effects of bosentan. Experiments were performed on 12-week-old male Wistar rats randomly assigned to 3 groups: control, monocrotaline-treated (60 mg/kg), and monocrotaline combined with bosentan (300 mg/kg/day). Our study confirmed the well-known effects of monocrotaline administration on lungs and the right ventricle, as well as pulmonary arterial pressure. In addition, we observed activation of the alternative pathway of the renin–angiotensin system, namely an increase in angiotensin (Ang) 1–7 and Ang 1-5 together with an increase in Ang I, but without any change in Ang II level, and downregulation of aldosterone 4 weeks after monocrotaline administration. For the first time, modifications of erythrocyte Na,K-ATPase enzyme kinetics were demonstrated as well. Our observations do not support data obtained in PAH patients showing an increase in Ang II levels, increase in oxidative stress, and deterioration in RBC deformability. Although bosentan primarily targets the vascular smooth muscle, our study confirmed its antioxidant effect. The obtained data suggest that besides the known action of bosentan, it decreases heart rate and increases erythrocyte deformability, and hence could have a beneficial hemodynamic effect in the PAH condition.

## 1. Introduction

Pulmonary hypertension is defined as a condition of increased mean blood pressure in pulmonary arteries to the value equal to or greater than 20 mmHg [1]. Pulmonary arterial hypertension (PAH) represents one of the pulmonary hypertension subtypes typical by multiple pathomechanisms leading to the clinical manifestation of the disease [2]. Despite being a rare disease, PAH deserves a continuous attention of the scientific community, considering the short survival time of PAH patients, even if treated. Complex pathogenesis is the cause of a variety of diverse treatment options that mainly target inflammation, endothelial function, proliferation of smooth muscle cells, and remodeling of the pulmonary arteries [3]. In the research on pathophysiological processes in PAH and for the identification of novel therapeutic strategies, a variety of animal models are being utilized. Preclinical models of PAH belonging to monocrotaline-treated rodents are frequently used. Monocrotaline induces endothelial cell damage resulting in pulmonary vascular remodeling, increased vascular resistance, and compensatory right ventricular hypertrophy ultimately leading to right heart failure and death [4].

Tissue remodeling is related to the biological activity of matrix metalloproteinases (MMPs), a family of zinc-dependent endopeptidases that degrade various proteins in the extracellular matrix. Their activity is regulated by four tissue inhibitors of matrix metalloproteinases (TIMPs), namely TIMP1-TIMP4. All MMPs are inhibited by TIMPs once they become activated, with the exception of MMP-2 and MMP-9, which can form complexes with TIMPs in their latent form. In the monocrotaline model of PAH, pulmonary activity and expression of MMPs, namely MMP-2 and MMP-9 and their endogenous inhibitor TIMP-1, were found to be increased [5,6]. In human PAH, the plasma MMP-2/TIMP-4 ratio was identified as a marker of disease severity as well as a prognostic factor [7]. The development and progression of PAH are also related to increased activation of the renin–angiotensin system. Plasma levels of angiotensin (Ang) II, as well as the ratio Ang II/Ang 1–7, were elevated in human PAH, suggesting reduced conversion of Ang II to Ang 1–7 [8].

It has been repeatedly shown that the properties of erythrocytes are impaired in various pathologies [9]. Reduced erythrocyte deformability and increased aggregation were observed in patients with PAH; in addition, erythrocyte aggregation increased with the disease progression [10]. Erythrocyte characteristics are largely dependent on the proper function of the sodium–potassium pump (Na,K-ATPase) in erythrocyte membranes. An early target in monocrotaline intoxication is the Na,K-ATPase in pulmonary arteries [11], but the effect of monocrotaline on the Na,K-ATPase function in erythrocyte membranes remains unclear. In addition, nitric oxide is a critical regulator of erythrocyte characteristics and deformability. Nitric oxide is also implicated as a signaling molecule in PAH pathogenesis. It was demonstrated that nitric oxide donors attenuated the elevation of right ventricular systolic pressure and pulmonary arterial thickening [12]. Further, it has been observed that nitric oxide synthase activity in erythrocytes of PAH patients was decreased [13]. Nevertheless, the effect of monocrotaline administration on erythrocyte-mediated nitric oxide production has not yet been fully elucidated.

Currently approved therapies for PAH are directed at the recognized abnormalities within the pulmonary vascular pathways. Bosentan was the first oral endothelin receptor antagonist effective in the treatment of PAH. In humans, the treatment effect of bosentan leads to a prolonged walking distance, and a reduction in the pulmonary artery pressure, and vascular resistance [14]. In monocrotaline-induced PAH, bosentan could reduce apoptosis and inflammation of endothelial cells [15] and decrease the mortality of experimental animals [16]. However, bosentan administration has been associated with a reduction in erythrocyte count, hematocrit, and hemoglobin concentrations in patients with heart failure [17]. Therefore, we assume that the effect of bosentan on erythrocyte quality in PAH is worth investigating.

The goal of our study was to contribute to the characterization of the monocrotaline-induced model of PAH, with emphasis on the renin–angiotensin–aldosterone system, parameters of oxidative stress, MMP activity, and erythrocyte parameters in blood samples of experimental animals. Moreover, we aimed to better elucidate the effect of bosentan treatment using the PAH animal model.

## 2. Results

### 2.1. Biometric Parameters

**Figure 1:**
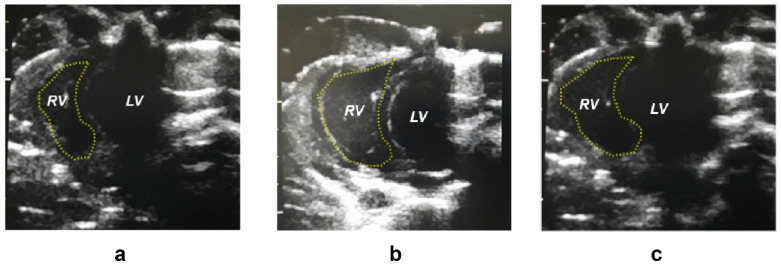
Representative images of the heart in diastole assessed by transthoracic echocardiography on days 20–21 after monocrotaline administration. Control group (**a**), monocrotaline-treated group (**b**), monocrotaline and bosentan-treated group (**c**). Abbreviations: LV—left ventricle, RV—right ventricle.

Monocrotaline administration resulted in a reduction in body weight (as well as normalized body weight adjusted to tibial length, *p* = 0.02) that was not affected by bosentan treatment. Neither monocrotaline administration nor bosentan treatment modified the systemic systolic blood pressure. Heart rate was reduced in the monocrotaline plus bosentan treated (BOS) group in comparison with the monocrotaline treated (MCT) group (*p* = 0.001). The right ventricle area was increased in the MCT group in comparison with control (CTRL) rats (*p* < 0.0001). Right ventricle wall thickness was higher in the MCT than in the CTRL group (*p* = 0.045) and bosentan treatment prevented this increase (*p* = 0.046) to a value similar to that in the CTRL group. Representative echocardiographic images are presented in Figure 1. The normalized right ventricular, left ventricular and septal weights were not modified after any intervention in the experimental animals. The difference in normalized right ventricular weight between the CTRL and the MCT groups with a *p*-value of 0.076 was noted as closest to statistical significance. Both systolic and mean pulmonary artery pressure were increased after MCT administration (*p* < 0.0001), but no differences were found between the MCT and BOS groups. Monocrotaline administration also led to an increase in the normalized lung (*p* = 0.001) and liver weight (*p* = 0.024). The measured values with significant differences between groups are presented in Table 1.

### 2.2. Angiotensin Peptide and Aldosterone Concentrations in Blood Plasma

Ang I, Ang 1–7, and Ang 1–5 levels were increased in MCT compared with the CTRL group (*p* = 0.007, 0.003, and 0.02). No additional significant differences in angiotensin plasma concentration were observed. Plasma aldosterone level decreased after monocrotaline administration when compared with the control rats (*p* = 0.002); however, this effect was prevented by bosentan treatment in the BOS group (*p* = 0.019) as can be seen in Table 2.

### 2.3. Parameters of Oxidative and Carbonyl Stress in Blood Plasma

No statistically significant differences between the groups were found in advanced glycation end products (AGEs) and thiobarbituric acid reactive substances (TBARS) plasma concentration. Bosentan treatment significantly lowered advanced oxidation protein products (AOPP) (*p* = 0.025) and fructosamine levels (*p* < 0.0001) when compared to the MCT group. Detailed values are presented in Table 3.

### 2.4. Parameters of Antioxidative Status in Blood Plasma

Plasma ferric reducing antioxidant power (FRAP) revealed no statistically significant differences between the studied groups (Table 3). BOS showed a decrease in total antioxidant capacity (TAC) compared with the MCT group with a *p*-value of 0.05. We found an increase (close to being statistically significant with *p* = 0.055) in plasma GSH concentration in the BOS group when compared with the MCT animals. This increase resulted from unchanged GSSG levels (*p* = 0.56) in a significantly higher ratio of reduced to oxidized glutathione (GSH/GSSG) in the BOS group (*p* = 0.037) in comparison with the MCT group (Table 3).

### 2.5. MMP-2, MMP-9 Activity, and TIMP-1 Concentrations in Blood Plasma

Neither monocrotaline administration nor bosentan treatment showed an effect on plasma activities of MMP-2, MMP-9, and the concentration of TIMP-1 as no significant differences among groups could be detected. Measured values are presented in Table 4.

### 2.6. Erythrocyte Parameters

Erythrocyte count was not affected by any of the given medications (*p* = 0.4). Monocrotaline administration reduced hematocrit in rats independently of the bosentan treatment (*p* = 0.02). Neither erythrocyte mean cell volume (MCV), nor the red blood cell distribution width (RDW-SD) were affected (MCV *p* = 0.21; RDW-SD *p* = 0.44). Monocrotaline alone did not affect erythrocyte deformability (*p* = 0.241), however, in the BOS group, this parameter was significantly higher when compared to MCT (*p* = 0.024). Regarding erythrocyte osmotic resistance (parameter IC_50_), no differences among groups were observed (*p* = 0.85). Similarly, nitric oxide production by erythrocytes was not altered by any of the given medications (*p* = 0.54). Measured values and significant differences between groups are presented in Table 5.

### 2.7. Na,K-ATPase Kinetics

**Figure 2:**
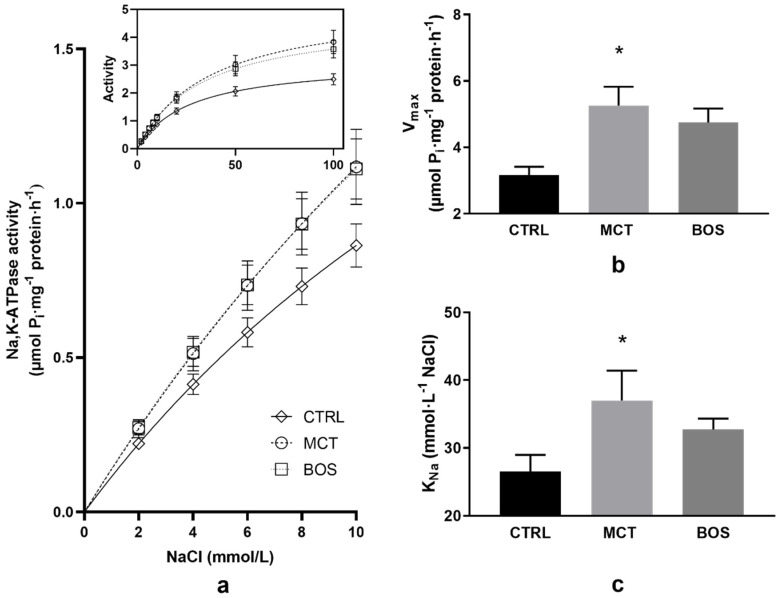
Activation of Na,K-ATPase in Na^+^ concentrations ranging from 2 to 100 mmol/L (**a**), kinetic parameters of Na,K-ATPase V_max_ (**b**), and K_Na_ (**c**) in erythrocyte membranes. Abbreviations: CTRL, control group; MCT, monocrotaline-treated group; BOS, monocrotaline- and bosentan-treated group; V_max_, maximal velocity of reaction; K_Na_, NaCl concentration required for half maximal activation of Na,K-ATPase, * *p* < 0.05 in comparison with CTRL group. Data are presented as means ± standard errors of mean, *n* = 9–11 per group.

Analysis of Na,K-ATPase activation with increasing concentration of NaCl revealed remarkable effects on erythrocytes of monocrotaline treated animals. The enzyme activity increased after monocrotaline administration throughout the investigated NaCl concentration range as compared with the CTRL group (Figure 2a). Data evaluation resulted in an increase of the V_max_ value by 67% with a significantly higher value of K_Na_ by 39% in the MCT group when compared with the controls (Figure 2b,c). Administration of bosentan to animals previously treated with monocrotaline revealed similar activities as in the MCT group throughout the concentration range investigated (Figure 2a). Evaluation of the data by nonlinear regression resulted in similar V_max_ and K_Na_ values in both MCT and BOS groups (Figure 2b,c).

## 3. Discussion

### 3.1. Biometry

In the present study, multiple effects of monocrotaline on basic biometric parameters were observed. Despite the normalized weight of the right ventricle increased only non-significantly, other findings, namely the increase in lung weight, right ventricle area, and wall thickness together with the elevated mean and systolic pulmonary pressure confirmed PAH in experimental subjects following monocrotaline administration. Regarding body weight, its decrease in PAH was recognized as an unfavorable prognostic factor. Lower body weight represents a strong risk factor for mortality in PAH pointing to the obesity paradox in human studies [18] as well as in the murine monocrotaline model of PAH [19]. The observed increase in liver weight after monocrotaline administration may be related to the structural remodeling of this organ. Monocrotaline can induce liver injury directly via the destruction of liver sinusoidal endothelial cells [20,21,22] or indirectly due to the development of the right ventricular hypertrophy and consequent liver congestion [23].

Available data show that bosentan treatment can lower mean pulmonary artery pressure both in humans and monocrotaline animal models [24,25]; however, in the present study, the pulmonary pressure-lowering potential of bosentan seems not that apparent. Although, bosentan administration probably prevented thickening of the right ventricle wall, which together with an attenuating effect on heart rate may indicate hemodynamic relief in the condition of PAH.

### 3.2. Renin–Angiotensin System Peptides and Aldosterone Plasma Concentration

Plasma Ang II concentrations have been shown to be higher in monocrotaline-induced PAH in Sprague–Dawley rats [26,27,28], and increased serum levels of renin, Ang I and Ang II are associated with progressive idiopathic PAH in humans [29]. Interestingly, an increase in plasma Ang I concentration observed in our study after monocrotaline administration did not result in a higher Ang II level. Instead, the promotion of alternative activity of the renin–angiotensin system was revealed as Ang 1–7 and Ang 1–5 increased simultaneously with Ang I elevation. An increase in Ang 1–7 after monocrotaline administration was also observed in the study of Falcão-Pires et al. [30], suggesting that its well-known vasodilatory properties could modify the excessive production of vasoconstrictors (e.g., Ang II and endothelin-1) and decrease right ventricle pressure overload. However, Ang 1–7 plasma levels have been shown to be decreased in PAH patients [31] and its increase might represent therapeutic potential in the monocrotaline model of PAH [32].

Aldosterone seems to contribute to monocrotaline-induced PAH development as its concentration was found to be increased in both plasma and lung homogenates, and spironolactone prevented pulmonary vascular remodeling [33]. Similar changes have been observed in human PAH [29]. These findings are contradictory to our observation of decreased aldosterone concentration after PAH induction. However, other authors have reported that either the transcripts or levels of natriuretic peptides were elevated in the condition of PAH [34,35,36,37]. These findings may provide an explanation for the surprisingly low plasma aldosterone level observed in our study simultaneously with unchanged Ang II concentration, as natriuretic peptides are inhibitors of aldosterone synthesis [38]. In other studies, bosentan administration normalized aldosterone levels at least partially due to its capability to reduce natriuretic peptides [39,40].

### 3.3. Oxidative Stress

Monocrotaline administration did not affect any of the investigated parameters of oxidative stress measured in blood plasma, although the published studies show increased lipid peroxidation and reduced antioxidant reserve in the lung tissue [41] as well as in erythrocytes [42] in monocrotaline-induced PAH. One may speculate that the tissue impairment was not sufficient enough to modify circulating oxidative parameters, or that analysis of other parameters may be more appropriate to reveal systemic oxidative stress following the monocrotaline administration.

Antioxidant effects of bosentan have already been demonstrated [43,44]. Changes in oxidative status were confirmed also by our study as bosentan treatment led to a reduced AOPP and fructosamine plasma concentrations and an improvement in plasma thiol-disulfide redox balance estimated via measurement of GSH/GSSG ratio in monocrotaline-treated animals.

### 3.4. MMPs

Increased activities of MMP-2, as well as TIMP-1, were documented in the pulmonary artery smooth muscle cells in patients with idiopathic PAH [45]. An increase in TIMPs in PAH represents a compensatory mechanism for the regulation of MMP activity; however, its effect seems to be limited, as the MMP-9/TIMP-1 ratio in lung tissue was also increased [46]. According to the literature, MMP-2 serum activity, as well as the gene expression were increased 4 weeks after monocrotaline injection [47]. However, despite the MMP-2, MMP-9, and TIMP-1 changes being documented in different tissues, in our experiment, pathologies in the lungs and heart were probably not sufficiently intense to alter MMP and TIMP plasma activities.

### 3.5. Erythrocyte Characteristics

A significant part of our experiment was focused on monitoring the erythrocyte properties. There are numerous indicators of erythrocyte involvement in the etiopathology of PAH. For example, hemolytic syndromes are associated with endothelial dysfunction as well as proliferative changes in the intima and media of blood vessels [48]. Hemolysis may play a significant role in PAH, as chronic exposure to low plasma hemoglobin concentrations induced pulmonary vascular disease via hemoglobin-mediated oxidation and inflammation in rodents [48]. In human PAH, an association between free hemoglobin concentration and the severity of PAH symptoms was established [49]. Regarding erythrocyte parameters, in the monocrotaline-induced model of PAH (2-4 mg/kg), an increase in hemoglobin concentration, hematocrit, and erythrocyte count was found [50,51]. On the other hand, another study with a higher monocrotaline dose (200 mg/kg) in mice did not report changes in hematocrit [52]. In our study, a decrease in hematocrit after monocrotaline administration independent of bosentan treatment was observed. This finding corresponds with the results of a human PAH study, revealing a decrease in hemoglobin, erythrocyte count, and hematocrit [10]. RDW-SD was suggested as a promising biomarker and prognostic factor of PAH [53]; however, there are no available data for RDW changes in the monocrotaline model of PAH. In our study, 4 weeks after monocrotaline injection, neither changes in RDW were present, nor erythrocyte osmotic resistance was affected.

In contrast to human PAH, no significant changes in erythrocyte deformability were observed four weeks after monocrotaline administration. Noteworthy, the transfusion of stored, less deformable erythrocytes led to an increase in mean pulmonary arterial pressure—i.e., transient PAH in humans and sheep [54,55]. These observations underlined the importance of erythrocyte quality in the condition of PAH. An increase in erythrocyte deformability after bosentan treatment in monocrotaline-treated animals may be related to improved hemodynamics. This effect is not entirely surprising, since bosentan treatment was also suggested as helpful for patients with sickle-cell anemia, as it prevented acute vasoocclusive crisis and reduced organ damage in the mice model of sickle cell disease [56]. This improvement is, at least partially, a consequence of its beneficial effect on the antioxidant defense of the organism that was also documented in our study. Concerning another erythrocyte pathology in PAH and nitric oxide metabolism in blood, a decrease in endothelial nitric oxide synthase activity and nitric oxide generation was reported in erythrocytes obtained from PAH patients [13]. In the monocrotaline model of PAH, serum nitric oxide level was not changed; however, the expression of nitric oxide synthase 3 mRNA in rat lung tissue was increased after monocrotaline administration and lowered after bosentan treatment [47]. In our study, nitric oxide metabolism of erythrocytes was not changed by any of the given medications, suggesting that nitric oxide is not involved in the observed changes in erythrocyte deformability. We also focused on bringing new data concerning the molecular principles of possible alterations of Na,K-ATPase in erythrocyte membranes of rats following the monocrotaline administration. The increase in Na,K-ATPase activities throughout the applied concentration range of NaCl was probably caused by an increased number of active enzyme molecules in erythrocyte membranes, as indicated by the higher value of V_max_. This may represent a compensatory effect to reduced hematocrit in the group of rats treated with monocrotaline. On the other side, the ability of the enzyme to bind sodium ions was impaired as indicated by increased K_Na_ value when compared to control rats. However, our finding of increased activity of Na,K-ATPase in erythrocytes of the MCT group, points to tissue specificity in the response of this enzyme to monocrotaline, as the enzyme activity was lowered after treatment with monocrotaline in myocardial tissue [57]. Our investigation of the effect of bosentan on the Na,K-ATPase activity in erythrocytes showed that in the alterations of the enzyme induced by monocrotaline, the endothelin is probably not involved. The similar Na,K-ATPase activities throughout the applied concentration range of NaCl indicate no alterations in Na-binding properties as confirmed also by similar values of K_Na_ in MCT and BOS groups.

## 4. Materials and Methods

### 4.1. Study Design

The experiments were performed on 12-week-old male Wistar rats, weighing 200–250 g (VELAZ, s.r.o., Prague, Czech Republic). Animals were housed at standard conditions: air temperature 22 ± 2 °C, relative air humidity of 55 ± 10%, light regime 12/12 h, suitable bedding, food, and drinking water ad libitum.

**Figure 3:**
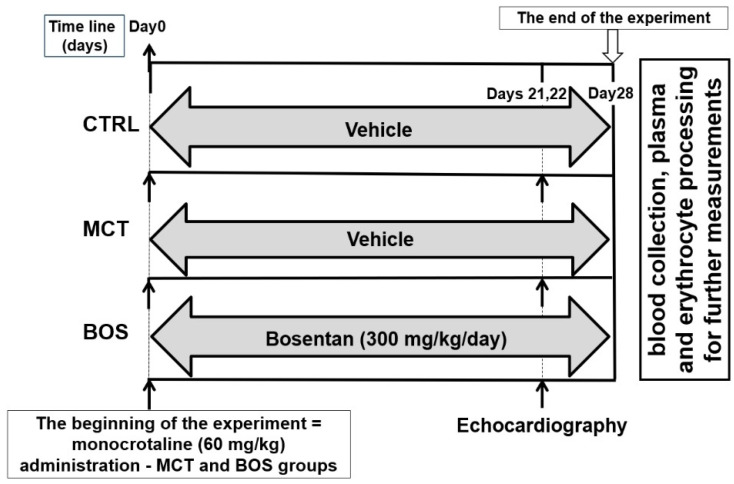
Experimental design. Abbreviations: CTRL, control group; MCT, monocrotaline-treated group; BOS, monocrotaline- and bosentan-treated group.

At the beginning of the experiment, 36 rats were randomly classified into 3 groups: control (CTRL), monocrotaline (MCT), and monocrotaline plus bosentan (BOS) treated group, with 12 animals in each group. As these animals were part of a larger experiment focused on new treatment options for PAH, all rats were intraperitoneally implanted with an osmotic minipump (2ML4 ALZET, Cupertino, CA, USA) with a pumping rate of 2.58 µL/h (61.92 µL/24 h) filled with saline (0.9% *w*/*v* i.v., B. Braun, Melsungen, Germany). During minipump implantation, monocrotaline was subcutaneously administered at the dose of 60 mg/kg to animals assigned to the MCT and the BOS groups, whereas the CTRL group received a corresponding volume of saline. The surgery was performed under 2.5–3% isoflurane-induced anesthesia in a highly sterile environment. Afterwards, the surgical glue was also applied to the sutured skin (RiverBand Surgical Adhesive 2 mL Skin Glue Tissue), the rats were housed individually and monitored for the next 48 h. The rats in the BOS group were treated with bosentan dissolved in drinking water at the dose of 300 mg/kg/day (according to [16]). The dissolved drug was administered once a day by oral gavage using a stainless-steel feeding probe with a rounded tip for 4 weeks following monocrotaline administration. To ensure equivalent experimental conditions in all animals, rats in the CTRL and the MCT groups were given drinking water (vehicle) in the corresponding volume by oral gavage. The experimental design is presented in Figure 3.

Due to the complexity of experimental conditions, the plasma levels of bosentan (as well as other drugs used in parallel, but not presented in this study) were measured at the end of the experiment in all experimental animals to check the accuracy of the treatment and to reveal the possible presence of an inappropriate drug in blood. One animal from the CTRL group, two from MCT, and two from BOS were excluded from the experiments due to drug contamination. Afterwards, bosentan concentration was 27.9 ± 8.89 µmol/L in the BOS group, but undetectable in the CTRL and MCT groups. One rat from the BOS group died on day 20. Finally, the following counts of experimental animals were used for all analyses: 11 in the CTRL, 10 in the MCT, and 9 in the BOS group.

Four weeks after monocrotaline administration, the rats were subjected to 2.5–3% isoflurane-induced anesthesia. Blood was obtained from the left heart ventricle to heparin-containing tubes (100 UI/mL). Hematocrit value, erythrocyte count, mean cell volume (MCV), as well as erythrocyte distribution width (RDW-SD) were determined by the blood analyzer (Sysmex F-820, Tokyo, Japan). Ten microliters of whole blood were also provided for the determination of nitric oxide production by erythrocytes. Afterwards, the remaining blood was centrifuged (10 min, 2000× *g*), and plasma was separated and stored at −80 °C until further analysis. The buffy coat and the uppermost layer of erythrocytes were removed. The remaining erythrocytes were washed three times in saline (0.9% NaCl) and used for the analysis of erythrocyte deformability and osmotic resistance, as well as for isolation of erythrocyte membranes and subsequent kinetic measurements of the Na,K-ATPase enzyme.

All procedures involving the use of experimental animals were performed in accordance with the Guide for the Care and Use of Laboratory Animals, 8th Edition (2010) published by the US Committee for the Update of the Guide for the Care and Use of Laboratory Animals; National Research Council, to the EU, adopted Directive 2010/63/EU of the European Parliament and the Council on the protection of animals used for experimental and other scientific purposes. The study was approved by the Ethical Committee of the Institute of Pathophysiology, Faculty of Medicine, Comenius University in Bratislava, Slovakia, and the State Veterinary and Food Administration of the Slovak Republic under No. 1067/19-221/3.

### 4.2. Pulmonary Artery Blood Pressure Measurements

Transthoracic echocardiography was performed in all animals on days 20–21 after monocrotaline administration. We used a 14 MHz matrix probe connected to the GE Medical Vivid 7 Dimension system (Prague, Czech Republic) in the short and long axis planes. Rats were anesthetized by a 2.5% inspiratory concentration of isoflurane (Isofluranum 1000 mg/g, Vetpharma Animal Health, Barcelona, Spain) at a flow rate of 2 L/min of O_2_ through a sealed nose cone during spontaneous breathing. Animals were placed on a heated pad in the supine position and shaved in the thoracic area. The heart rate, as well as body temperature, were monitored continuously. Mean pulmonary artery blood pressure was derived according to Urboniene et al. [58] based on the measurement of the time integral of the pulmonary valve blood flow velocity and pulmonary artery acceleration time.

### 4.3. Angiotensin Peptide Concentration Assessment

The concentration of angiotensin peptides (Ang I, Ang II, Ang III, Ang IV, Ang 1–7, and Ang 1–5) and aldosterone in blood plasma was determined by Attoquant Diagnostics as described previously [59]. The conditioned heparinized plasma was incubated for 60 min at 37 °C and stabilized. Samples were then spiked with 200 pg/mL of a stable isotope-labelled internal standard for each angiotensin metabolite. Following C18-based solid-phase-extraction, samples were analyzed by liquid chromatography–tandem mass spectroscopy (LC-MS/MS) using a reversed-phase analytical column (Acquity UPLC^®^ C18, Waters Corp., Milford, MA, USA) operating in line with a XEVO TQ-S triple-quadrupole mass spectrometer (Waters Corp.) in MRM mode. In house standards were used to correct for peptide recovery of the sample preparation procedure for each angiotensin metabolite. Calculations of angiotensin peptide levels were performed considering the corresponding response factors determined in the fitting calibration curves in the original sample matrix, on the condition that integrated signals exceed a signal-to-noise ratio of 10 [59].

### 4.4. Parameters of Oxidative Stress and Antioxidant Status in Blood Plasma

Markers of oxidative damage and antioxidant status were assessed in plasma samples. Measurements were performed using a Synergy H1 Hybrid Multi-mode Reader (Agilent, Santa Clara,, CA, USA). All chemicals used in the analysis were purchased from Sigma-Aldrich (Steinheim, Germany). If not stated otherwise, we proceeded as described in our previous study [60].

Measurement of advanced oxidation protein products (AOPP) was used to determine the protein oxidative damage. Lipid peroxidation was estimated by measuring thiobarbituric acid reactive substances (TBARS). Regarding the marker of carbonyl stress, advanced glycation end products (AGEs) and fructosamine concentrations were determined. The concentrations of AOPP, AGEs, and fructosamine were adjusted to plasma protein concentrations determined by means of the Bicinchoninic Acid Kit according to the manufacturer’s instructions (Sigma-Aldrich, Steinheim, Germany), with bovine serum albumin used as a standard.

The ratio of reduced to oxidized glutathione (GSH/GSSG) was used as a general marker of oxidative stress. Measurements of ferric reducing antioxidant power (FRAP) and total antioxidant capacity (TAC) were used as markers of antioxidant status. TAC measurements were carried out as described previously [61].

### 4.5. MMP-2 and MMP-9 Activity and TIMP-1 Level in Blood Plasma

Activities of the gelatinases MMP-2 and MMP-9 were assessed by the substrate-zymography technique [60]. It is a semi-quantitative method that uses different types of substrates allowing the detection of MMPs activity. The activities of circulating MMP-2 and MMP-9 in plasma samples were determined by gelatin–zymography in 10% polyacrylamide gel, containing gelatin (2 mg/mL) as a substrate. The MMPs were separated by electrophoresis under denaturing, but not reducing conditions. After separation, the gels were washed twice for 20 min (50 mmol/L Tris-HCl, pH 7.4, containing 2.5% Triton X-100), and then incubated overnight at 37 °C in an appropriate “refolding” buffer containing Ca^2+^ ions (50 mmol/L Tris-HCl, pH 7.4, 10 mmol/L CaCl_2_ and 1.25% Triton X-100). Afterward, the gels were stained with 1% Coomassie Brilliant Blue G-250 for 3 h. Subsequent destaining with 40% methanol and 10% acetic acid solution revealed the zone of lysis as a clear region (band) on a uniform blue background. The MMP activities were quantified by densitometry according to the area of the corresponding bands. The pixel density of each band was detected using ImageJ analysis software (NIH, Bethesda, MD, USA).

TIMP-1 was determined using the Rat Cytokine Array Q67 kit (RayBiotech, Peachtree Corners, GA, USA) according to the manufacturer’s instructions. TIMP-2 was also supposed to be determined using this kit, but its concentrations were below the detection limit in the vast majority of our plasma samples.

### 4.6. Erythrocyte Deformability, Nnitric Oxide Production, and Osmotic Resistance

To evaluate erythrocyte deformability, erythrocytes were washed and diluted in the Cellpack solution (diluent for Sysmex blood analyzer, 1:1000, *v/v*) followed by filtration using centrifugation (at 175× *g*) through membrane filters with 5 μm diameter pores (Ultrafree-MC SV Centrifugal Filter; Merck Millipore Ltd., Tullagreen Carrigtwohill, Ireland). Deformability of erythrocytes was calculated as the ratio of erythrocyte count after the filtration to erythrocyte count before the filtration.

For measurement of nitric oxide production by erythrocytes, the fluorescent 4,5-diaminofluorescein diacetate (DAF-2 DA, Abcam, 25 μmol/L) was used. Whole blood was diluted 1:9 (v:v) in phosphate-buffered saline and incubated with DAF2-DA for 10 min at room temperature in the dark. In each sample, the fluorescence of approximately one thousand erythrocytes was determined using the fluorescence microscope (Axio Imager A2, Zeiss, Germany) and ImageJ software (NIH, Bethesda, MD, USA).

For erythrocyte osmotic resistance determination, washed erythrocytes were suspended in a series of solutions with ascending NaCl concentrations ranging from 0.1 to 0.9%. After a 30-min incubation at room temperature, suspensions were centrifuged (1200× *g*, 5 min) and the degree of hemolysis in supernatants was determined spectrophotometrically at 540 nm. Absorbance obtained at 0.9% NaCl was adjusted to non-hemolytic (0% of hemolysis) and absorbance value at 0.1% NaCl referred to total (100%) hemolysis. Measured data were interpolated to a sigmoidal curve, and the NaCl concentration at which 50% hemolysis occurred (IC_50_), was determined.

Erythrocyte deformability, nitric oxide production, and osmotic resistance were assessed as described elsewhere [62].

### 4.7. Kinetic Measurements of Na,K-ATPase in Erythrocyte Membranes

To isolate erythrocyte membranes, washed erythrocytes were mechanically homogenized and all their intracellular components were washed away in a series of hypotonic solutions of TRIS as in our previous study [63]. The protein concentration in each sample was determined by Lowry’s method using bovine serum albumin as a standard [64]. The Na,K-ATPase activities were determined in the range of NaCl concentrations (2–100 mmol/L) as specified previously [65]. The measured data were analyzed by nonlinear regression and used to evaluate kinetic parameters: maximum velocity of enzyme reaction (V_max_), and concentration of Na^+^ necessary for half-maximal activation of the enzyme (K_Na_).

### 4.8. Statistical Analyses

Data are presented as mean ± standard deviation when normally distributed or as median with interquartile range if the distribution was non-Gaussian. The normality of data was determined by the Shapiro–Wilk test. One-way ANOVA with Dunnett’s multiple comparisons test (or Kruskal–Wallis test with Dunn’s multiple comparisons test in the case of non-Gaussian distribution of data) was used to reveal the differences among the experimental groups. In the multiple comparison tests, the CTRL and the BOS groups were compared to the MCT group. For statistical analysis and graphical illustration of data, GraphPad Prism 8 and SigmaPlot 13 software were used. Differences with *p* < 0.05 were considered statistically significant.

## 5. Conclusions

**Figure 4:**
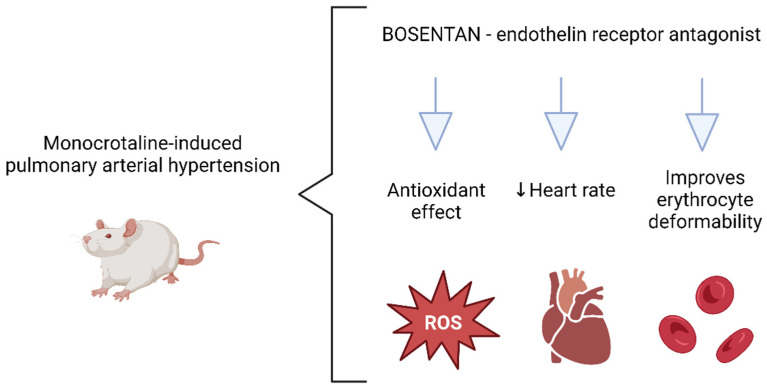
The effects of bosentan treatment in the pulmonary arterial hypertension induced by monocrotaline administration in Wistar rats.

Our study demonstrates the additional effects of monocrotaline: activation of the alternative pathway of the renin–angiotensin system, downregulation of aldosterone, and modifications in erythrocyte Na,K-ATPase kinetics in a monocrotaline-induced rat model of PAH. In our rat model, bosentan, as one of the compounds approved for PAH treatment in humans, showed an antioxidant effect, decreased heart rate, and increased erythrocyte deformability (Figure 4); hence, we suppose that it contributes to the improvement of hemodynamics in the condition of PAH.

## Figures and Tables

**Table 1:** Selected biometric parameters of experimental animals.

	CTRL (*n* = 11)	MCT (*n* = 10)	BOS (*n* = 9)
**Body weight (g)**	431 ± 17.4 *	408 ± 21.5	412 ± 17.6
**Blood pressure (mmHg)**	139 ± 5.61	143 ± 7.05	139 ± 7.65
**Heart rate (min^−1^)**	380 ± 29.1	408 ± 36.3	347 ± 39.3 **
**Right ventricle area d (mm^2^)**	28.0 ± 5.26 ****	48.3 ± 5.77	44.3 ± 7.55
**Right ventricle wall thickness (mm)**	1.34 ± 0.39 *	1.70 ± 0.14	1.34 ± 0.15 *
**Right ventricle/body weight (g/kg)**	0.46 ± 0.11	0.65 ± 0.22	0.63 ± 0.27
**PASP (mmHg)**	12.9 ± 8.87 ****	60.4 ± 11.7	58.5 ± 9.99
**mPAP (mmHg)**	13.1 ± 3.25 ****	30.6 ± 4.33	29.8 ± 3.67
**Lungs/body weight (g/kg)**	4.27 ± 0.36 ***	5.20 ± 0.56	5.31 ± 0.59
**Liver/body weight (g/kg)**	36.5 ± 1.89 *	39.1 ± 1.39	38.6 ± 3.04

Data are presented as means ± standard deviations. Abbreviations: CTRL, control group; MCT, monocrotaline-treated group; BOS, monocrotaline- and bosentan-treated group, PASP, pulmonary artery systolic pressure; mPAP, mean pulmonary artery pressure. Statistical significance: * *p* < 0.05, ** *p* < 0.01, *** *p* < 0.001, **** *p* < 0.0001 in comparison with the MCT group.

**Table 2:** Angiotensin peptide and aldosterone concentrations in blood plasma.

	CTRL (*n* = 11)	MCT (*n* = 10)	BOS (*n* = 9)
**Ang I (1–10) (pmol/L)**	398 ± 142 **	738 ± 265	576 ± 266
**Ang II (1–8) (nmol/L)**	0.80 ± 0.26	1.08 ± 0.43	1.04 ± 0.24
**Ang 1–7 (pmol/L)**	11.9 ± 2.92 **	26.3 ± 11.3	19.1 ± 7.25
**Ang 1–5 (pmol/L)**	34.4 ± 11.7 *	58.6 ± 26.6	42.4 ± 15.6
**Ang III (2–8) (pmol/L)**	38.5 ± 12.6	51.6 ± 20.8	50.9 ± 12.0
**Ang IV (3–8) (pmol/L)**	44.6 ± 12.3	60.7 ± 20.4	55.0 ± 13.0
**Aldosterone (nmol/L)**	0.51 (0.31; 1.22) **	0.10 (0.08; 0.20)	0.51 (0.16; 0.92) *

Data are presented as means ± standard deviations for parametric data or as the median and interquartile range for non-parametric data. Abbreviations: CTRL, control group; MCT, monocrotaline-treated group; BOS, monocrotaline- and bosentan-treated group; Ang, angiotensin. Statistical significance: * *p* < 0.05, ** *p* < 0.01 in comparison with the MCT group.

**Table 3:** Plasma markers of oxidative and carbonyl stress and antioxidative status.

	CTRL (*n* = 11)	MCT (*n* = 10)	BOS (*n* = 9)
AGEs (g/g protein)	0.19 ± 0.06	0.20 ± 0.07	0.15 ± 0.04
AOPP (µmol/g protein)	8.53 ± 4.93	12.4 ± 9.80	4.51 ± 1.99 *
Fructosamine (mmol/g protein)	0.56 ± 0.06	0.66 ± 0.14	0.36 ± 0.12 ****
TBARS (µmol/L)	74.1 ± 17.5	101 ± 19.5	97.7 ± 43.4
FRAP (µmol/L)	201 ± 42.6	204 ± 44.1	187 ± 16.2
TAC (mmol/L)	4.43 ± 0.42	4.58 ± 0.26	4.20 ± 0.38
GSH/GSSG	58.8 ± 22.1	62.1 ± 38.3	118 ± 75.7 *

Data are presented as means ± standard deviations. Abbreviations: CTRL, control group; MCT, monocrotaline-treated group; BOS, monocrotaline- and bosentan-treated group; AGEs, advanced glycation end products; AOPP, advanced oxidation protein products; TBARS, thiobarbituric acid reactive substances; FRAP, ferric reducing antioxidant power; TAC, total antioxidant capacity of plasma; GSH, reduced glutathione GSSG, oxidized glutathione. Statistical significance: * *p* < 0.05, **** *p* < 0.0001 in comparison with the MCT group.

**Table 4:** MMP-2 and MMP-9 activities, and TIMP-1 concentrations in blood plasma.

	CTRL (*n* = 11)	MCT (*n* = 10)	BOS (*n* = 9)
MMP-2 (a.u.)	7.97 ± 1.09	8.11 ± 2.26	6.52 ± 1.37
MMP-9 (a.u.)	3.09 ± 0.93	2.81 ± 0.92	2.56 ± 1.34
TIMP-1 (ng/mL)	1.98 ± 0.26	1.89 ± 0.19	1.88 ± 0.15

Data are presented as means ± standard deviations. Abbreviations: CTRL, control group; MCT, monocrotaline-treated group; BOS, monocrotaline- and bosentan-treated group; MMP, matrix metalloproteinase; TIMP, tissue inhibitor of MMPs; a.u.—arbitrary unit.

**Table 5:** Erythrocyte parameters.

	CTRL (*n* = 11)	MCT (*n* = 10)	BOS (*n* = 9)
Count (10^9^/mL)	7.49 ± 0.42	7.25 ± 0.47	7.17 ± 0.28
Hematocrit (%)	43.8 ± 3.99 *	39.6 ± 1.74	39.5 ± 2.34
Mean Cell Volume (fL)	56.0 ± 2.10	55.5 ± 1.12	56.0 ± 0.70
RDW-SD (fL)	27.7 ± 1.50	26.8 ± 1.01	27.1 ± 1.11
Deformability (%)	37.0 ± 3.65	32.1 ± 7.08	40.5 ± 5.24 *
IC_50_ (% NaCl)	0.46 ± 0.02	0.46 ± 0.01	0.46 ± 0.02
Nitric Oxide Production (a.u.)	9.93 ± 2.22	8.58 ± 2.68	9.33 ± 2.29

Data are presented as means ± standard deviations. Abbreviations: RDW-SD, red cell distribution width; IC_50_, NaCl concentration, in which 50% hemolysis occurred; a.u., arbitrary unit, CTRL, control group; MCT, monocrotaline-treated group; BOS, monocrotaline- and bosentan-treated group. Statistical significance: * *p* < 0.05 in comparison with the MCT group.

## Data Availability

Data is contained within the article.
